# Editorial: The role of immune checkpoints in gastrointestinal diseases

**DOI:** 10.3389/fphys.2023.1251966

**Published:** 2023-07-31

**Authors:** Xiaofei Shen, Junfeng Du

**Affiliations:** ^1^ Division of Gastric Surgery, Department of General Surgery, Nanjing Drum Tower Hospital, The Affiliated Hospital of Nanjing University Medical School, Nanjing, China; ^2^ Department of General Surgery, The 7th Medical Center, Chinese PLA General Hospital, Beijing, China

**Keywords:** immune checkpoint inhibitor (ICI), treatment efficacy, gastrointestinal disease, immunotherapy, gut microbiota

Immunotherapy is one of the most cutting-edge fields in the current treatment of gastrointestinal (GI) diseases (Abdul-Latif et al., 2020). Immune checkpoint inhibitors (ICIs), as one of the most recognized immunotherapy strategies, have been gradually applied in the clinical treatment of GI tumors and have also been gradually explored in GI inflammatory diseases. However, whether ICIs can be used under the circumstances of most digestive diseases, and how to combined use of ICIs with other therapeutic drugs has still lack of clinical evidence. In addition, accumulating evidence has shown that unlike the success of cancer immunotherapies in certain cancer types like melanoma (Larkin et al., 2015), the overall response rate (ORR) of ICIs therapy in the non-selective GI patients is still not satisfactory even though these patients may have been predicted to be responsive based on the expression levels of molecules such as PD-L1 (Ganesh et al., 2019). Therefore, how to improve current prediction strategies so as to benefit more patients based on the expression patterns of immune checkpoints on different immune cells has also been an important research field. In this Research Topic, with the efforts of five guest editors, 12 articles consisting of 6 original researches, 4 reviews, and 2 case reports were collected, providing a deep understanding and new comprehensive insights of the application of immunotherapy in gastrointestinal diseases, especially in gastrointestinal cancers. These findings partly help to answer questions mentioned above in the research field of “*The role of immune checkpoints in gastrointestinal diseases*.”

Most of the studies in this Research Topic were related to cancer process. Esophageal cancer (EC) is one of the deadliest malignancies due to its late-stage diagnosis, and immunotherapies, represented by ICIs, has gained promising perspectives for the treatment of patients with EC (Wadhwa et al., 2023). There is a lack of adequate evidence for the application of immunotherapies in treating patients with locally advanced EC. Qin et al. carried out a comprehensive meta-analysis to compare the efficacy and safety of the neoadjuvant use of ICIs combined with chemotherapy or chemoradiotherapy. Their results indicated that neoadjuvant immunotherapy could significantly improve the prognosis of patients with locally advanced EC, with acceptable toxicity. With regard to those with initially unresectable locally advanced EC, Huang et al. performed a real-world clinical trial and found that immunotherapy can offer patients a chance to receive a radical resection. Conversion surgery following immunochemotherapy was feasible and safe for these patients, with a better radiological and pathological response.

How about results on the application of ICIs in other locally advanced GI cancer? As one of the most common malignant tumors over the world, treatment strategy involving ICIs has already started in CRC, which has shown favorable outcomes against deficient mismatch repair (dMMR)/high levels of microsatellite instability (MSI-H) CRC (Schurch et al., 2020). Yang et al. reviewed recent findings about above achievements and proposed that adding immunotherapy into neoadjuvant therapy may change the treatment strategy of primary resectable or some metastatic CRC to reduce clinical stage but also to benefit patients to achieve a better local control. To test this hypothesis, the same group conducted a prospective, single-arm trial of long-course chemoradiotherapy combined with concurrent tislelizumab in locally advanced rectal cancer, to explore the safety and efficacy. Their results showed that long-course chemoradiotherapy combined with concurrent tislelizumab in patients with locally advanced low rectal cancer had favorable safety and efficacy, and did not increase the complication rate of surgery. Similar to these results, Chen et al. reviewed completed and ongoing clinical trials with ICIs in the area of gastroesophageal cancer (GEC). They found that ICIs combined with chemotherapy can be an effective first-line treatment and a monotherapy in second-line or more treatment and in maintenance therapy. To achieve a better response, Chen et al. also suggested that current biomarkers for predicting ICIs efficacy should be improved.

In consistent with above notion, one research group explored the expression patterns of immune checkpoints on cancer tissue and peripheral blood T cells in patients with gastric cancer. They found that the expression levels of immunosuppressive markers were significantly increased in cancer tissues and peripheral blood T cells, suggesting that peripheral blood analysis may be an important tool for prognostic assessment of patients with gastric cancer. Based on the co-expression of immune checkpoint molecules on T cells, does combined use of immune checkpoint inhibitors represent a potential promising strategy to improve current efficacy of ICIs? In one case report study, Peng et al. explored this aspect and found that patients with HER-2-positive advanced gastroesophageal junction cancer received PD-1/CTLA-4 bispecific immunotherapy combined with chemotherapy could achieve a complete remission.

Despite above aspects about the application of ICIs in treating GI cancers and strategies to predict and improve the efficacy of ICIs, toxicity is also a major problem to limit the use of ICIs (Tang et al., 2021). Zhou et al. reviewed findings about the adverse events of ICIs, especially for ICI-related colitis. They proposed that the gut microbiota acted as an important regulator in the pathogenesis of ICI-related colitis, and microbiota modulations like probiotics and fecal microbiota transplantation might be potential therapeutic strategy to treat these adverse events of ICIs.

In summary, the 12 articles in this Research Topic explore or discuss the application of ICIs in treating GI diseases, and provide potential strategies to predict and/or improve the efficacy of ICIs. Based on the importance of gut microbiota in predicting the efficacy of ICIs and their regulation in ICI-related adverse events (Lu et al., 2022), more insightful studies on the role and regulatory mechanisms of gut microbiota in participating ICIs responses are urgently needed, which may provide more promising therapeutic strategies in this area.
